# Esophagogastroduodenoscopy in Patients Aged 75 Years and Older: A Single-Center Study

**DOI:** 10.7759/cureus.21846

**Published:** 2022-02-02

**Authors:** Muhammer Ergenç, Tevfik Kıvılcım Uprak

**Affiliations:** 1 General Surgery, Istanbul Sultanbeyli State Hospital, Istanbul, TUR; 2 General Surgery, Marmara University School of Medicine, Istanbul, TUR

**Keywords:** octogenarians, aged, gastrointestinal endoscopy, digestive system, gastroscopy, esophagogastroduodenoscopy

## Abstract

Introduction: Esophagogastroduodenoscopy is frequently used for the elderly population. Older patients are more fragile than younger patients because of multiple age-related chronic diseases and the common use of polypharmacy. There is no adequate data in the existing literature regarding the application of upper gastrointestinal system endoscopy in the elderly population. Therefore, in this article, we evaluated esophagogastroduodenoscopy procedures that were performed on patients aged 75 years or older in the secondary care hospital.

Methods: We performed a retrospective observational study of patients aged 75 years or older who underwent esophagogastroduodenoscopy between January 2016 and January 2021 at the Istanbul Sultanbeyli State Hospital Endoscopy Unit. Indications of endoscopy, ages, genders, endoscopic diagnoses, polyp/tumor/biopsy localizations, histopathological examination of biopsies, and complications of esophagogastroduodenoscopy were analyzed.

Results: A total of 202 patients were analyzed. The most common indication was dyspepsia (25%), followed by gastrointestinal bleeding, reflux, anemia, and screening/surveillance. For patients aged 75-79 years and patients aged ≥80 years, endoscopic diagnoses of esophageal and gastric malignancies were observed as 6.4% and 18%, respectively. Very relevant findings of endoscopy (esophageal and gastric malignancies; gastric and duodenal ulcers) were detected in 39 (19.3%) of all included patients. No complications due to endoscopic procedures were observed, but complications due to sedation (hypotension and hypoxemia) were observed in 5.0%.

Conclusion: After pre-procedural evaluation, we must be careful while doing endoscopic procedures in the elderly because of multiple age-related chronic diseases and the common use of polypharmacy. This present study showed that esophagogastroduodenoscopy is a safe procedure with a high diagnostic yield in patients aged 75 years and older.

## Introduction

Although diseases and injuries occur regularly, the world population continues to live longer. The average life expectancy (73.3 years) and healthy life expectancy (63.7 years) in 2019 showed marked increases since 2000. With that increasing age, the incidence of gastrointestinal (GI) diseases in the elderly increased as well [1].

GI endoscopy is one of the most important tools for diagnosis, surveillance, and treatment of GI diseases. Endoscopic procedures have been applied more and more frequently in the elderly. The published data shows that esophagogastroduodenoscopy (EGD) is high-yielding in elderly patients, though we must take care to apply the procedure safely because of this age group’s frailty. Symptoms of anemia, dysphagia, epigastric pain, and GI bleeding, especially in male patients, should be taken into consideration for EGD because of increased malignancy or peptic ulcer disease risk [2,3].

We need more information about the application of upper GI system endoscopy in the elderly population. Therefore, in this article, we evaluated EGD procedures that were performed on patients aged 75 years or older in the Istanbul Sultanbeyli State Hospital. The aim of this study was to analyze the indications, endoscopic diagnosis, histopathological findings, and complications of upper GI endoscopy.

## Materials and methods

We performed a retrospective observational study of patients who underwent EGD from January 2016 to January 2021 at the Istanbul Sultanbeyli State Hospital Endoscopy Unit. This study was approved by the Marmara University Faculty of Medicine Clinical Research Ethics Committee (Number: 09.2021-724) and registered with ClinicalTrials.gov (NCT05012527).

We used patients’ endoscopy and hospital records for data acquisition. Patients with missing data and duplicate records were excluded from the study. Patients aged 75 years or older who underwent EGD were included in the analysis. The following parameters were analyzed: age, gender, endoscopy indications, endoscopic diagnosis, polyp/tumor/biopsy localization, histopathological examination of biopsies, and complications. For further analysis, patients were stratified according to age into two groups: 75-79 and ≥80. The endoscopy indications were classified by the American Society for Gastrointestinal Endoscopy (ASGE) guidelines [4]. Morphological features and grade of endoscopic gastric biopsies were reported according to the Sydney System (*Helicobacter pylori*/atrophy/intestinal metaplasia/chronic inflammation/activity) [5]. Malignancy, esophagitis, esophageal varices, gastritis, gastric erosion, gastric ulcer, gastric polyp, duodenitis, duodenal ulcer, and duodenal polyp are defined as relevant endoscopic findings. Esophageal malignancy, gastric ulcer, gastric malignancy, and duodenal ulcer are defined as very relevant endoscopic findings [6]. We calculated the overall diagnostic yield according to these parameters.

All endoscopies were performed using standard video-endoscopes (Fujinon EG-530WR) by seven general surgeons who had at least five years of experience in endoscopy. The patients fasted for at least 8 hours before the procedure. Almost all EGD procedures were performed while the patients were under sedation. Conscious sedation was achieved with propofol 1% 10 mg/ml by an anesthetic technician under the supervision of an anesthesiologist. Continuous monitoring was provided by recording oxygen saturation, blood pressure, and pulse rate.

The primary outcome of this study was to determine indications, endoscopic diagnosis, histopathological findings, and complications of upper GI endoscopy.

Statistical analysis

We performed statistical analysis using the Statistical Package for Social Sciences (Version 24 for Mac, IBM Corporation, Armonk, New York, USA). Chi-square and Fisher exact tests were used to compare categorical variables. For quantitative variables, a t-test, a Mann-Whitney U-test, a Kruskal-Wallis test, and an analysis of variance (ANOVA) were applied. P values less than 0.05 were considered statistically significant.

## Results

**Figure 1:**
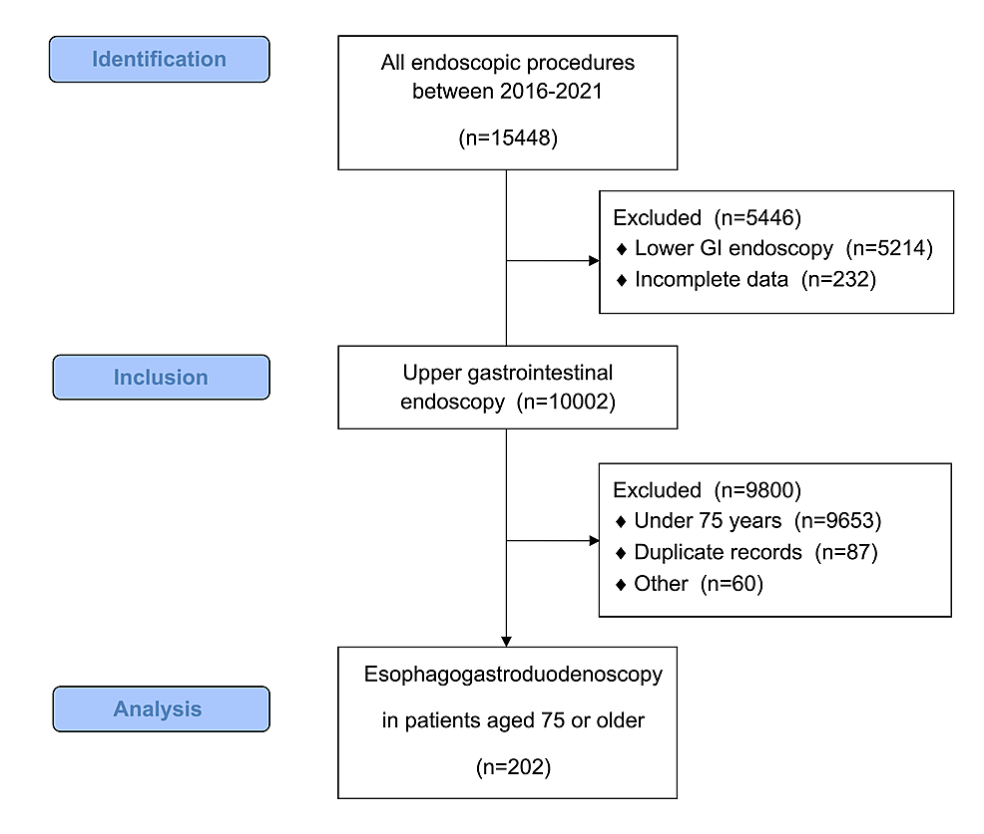
Flowchart of patient selection GI: gastrointestinal

A total of 202 patients were evaluated retrospectively (Figure 1).

Seventy-one of the patients were male (35%). The mean age was found to be 79 years. When the endoscopy indications were examined, the most common indication was dyspepsia (25%), followed by GI bleeding, reflux, anemia, and screening/surveillance.

Endoscopic findings were examined, and antral gastritis and pangastritis were observed frequently. The incidence of malignancy for all patients ranged from 2% for the esophagus and up to 9% for the stomach. The prevalence of esophageal and gastric malignancies in patients over 80 years of age was observed as 3.9% and 14.3%, respectively. The pathological findings showed that chronic active gastritis was found in 42.6% of the patients. The final diagnoses of some cases that were reported by endoscopists as malignancy were found to be premalignant lesions in pathology. GI system malignancy detected pathologically was 9.4% in the entire cohort and 15% in the patients over 80 years of age. However, this difference was not statistically significant (p = 0.24) (Table 1).

**Table 1:** Patients’ demographics, indications, and endoscopic and pathological findings GI: gastrointestinal; VRF: very relevant findings (esophageal malignancy, gastric ulcer, gastric malignancy, and duodenal ulcer)

Parameters	Total n: 202 (%)	Age group 75-79, n	Age group ≥80, n	P-value
Age (years, mean-range)	79			
Sex				0.98
Female	131	81	50	
Male	71	44	27	
Endoscopy indications				0.48
Dyspepsia	52 (25.7%)	34	18	
Abnormal imaging	10 (5.0%)	8	2	
Reflux	21 (10.4%)	12	9	
Dysphagia	11 (5.4%)	5	6	
Upper GI bleeding	25 (12.4%)	13	12	
Anemia	21 (10.4%)	11	10	
Vomiting	10 (5.0%)	6	4	
Screening/surveillance	21 (10.4%)	13	8	
Weight loss	13 (6.4%)	8	5	
Abdominal pain	18 (8.9%)	15	3	
Endoscopic diagnosis				0.07
Esophagitis	15 (7.4%)	11	4	
Bile reflux gastritis	4 (2.0%)	1	3	
Duodenal ulcer	4 (2.0%)	2	2	
Duodenal polyp	2 (1.0%)	0	2	
Normal finding	19 (9.4%)	16	3	
Gastric ulcer	13 (6.4%)	7	6	
Duodenitis	9 (4.5%)	6	3	
Gastric polyp	10 (5.0%)	7	3	
Other	5 (2.5%)	4	1	
Esophageal malignancy	4 (2.0%)	1	3	
Esophageal varices	3 (1.5%)	1	2	
Hiatal hernia	15 (7.4%)	8	7	
Antral gastritis	27 (13.4%)	21	6	
Pangastritis	24 (11.9%)	17	7	
Gastric erosion	16 (7.9%)	9	7	
Gastric malignancy	18 (8.9%)	7	11	
Findings secondary to operation	14 (6.9%)	7	7	
Very Relevant Findings (VRF)	39 (19.3%)	17 (13.6%)	22 (28%)	0.008
Pathological Finding				0.24
Chronic active gastritis	86 (42.6%)	54	32	
Low-grade dysplasia	3 (1.5%)	2	1	
Mucinous carcinoma	2 (1.0%)	0	2	
Inflammatory fibroid polyp	1 (0.5%)	0	1	
Chronic gastritis	65 (32.2%)	45	20	
Adenocarcinoma	17 (8.4%)	7	10	
Hyperplastic polyp	6 (3.0%)	4	2	
Ulceration	4 (2.0%)	2	2	
Duodenitis	1 (0.5%)	1	0	
Chronic esophagitis	2 (1.0%)	0	2	
Erosive gastritis	3 (1.5%)	2	1	

There was no significant difference between age groups in terms of pathological results (Table 2).

**Table 2:** Comparison of pathologic outcomes by age groups

Parameters	Total n: 202 (%)	Age group 75-79, n	Age group ≥80, n	P-value
Atrophy				0.9
None	147 (91%)	98	49	
Mild	10 (6%)	7	3	
Moderate	5 (3%)	3	2	
Helicobacter pylori				0.45
None	36 (22.4%)	27	9	
Mild	93 (57.8%)	60	33	
Moderate	32 (19.9%)	20	12	
Intestinal metaplasia				0.58
None	127 (78.4%)	88	39	
Mild	18 (11%)	10	8	
Moderate	15 (9.3%)	9	6	
Severe	2 (1.2%)	1	1	
Chronic inflammation				0.191
Mild	110 (68%)	77	33	
Moderate	52 (32%)	31	21	
Activity				0.38
None	75 (46.3%)	52	23	
Mild	70 (43.2%)	47	23	
Moderate	16 (9.9%)	9	7	
Severe	1 (0.6%)	0	1	

A positive correlation was found between atrophy and intestinal metaplasia, while a negative correlation was found with the density of *H. pylori* (p = 0.00 and p = 0.04, respectively). The density of *H. pylori* was significantly correlated with the severity of chronic inflammation and activity (p = 0.00 and p = 0.00, respectively). On the other hand, there was a significant positive correlation between intestinal metaplasia and chronic inflammation (p = 0.005) (Table 3).

**Table 3:** Correlations between pathologic outcomes *Correlation is significant at the 0.05 level (Two-tailed) **Correlation is significant at the 0.01 level (Two-tailed)

	Atrophy	Helicobacter pylori	Intestinal metaplasia	Chronic inflammation	Activity
Atrophy	1.000	−0.157^*^	0.616^**^	0.054	0.151
	.	0.046	0.000	0.495	0.055
Helicobacter pylori	−0.157^*^	1.000	−0.061	0.453^**^	0.431^**^
	0.046	.	0.439	0.000	0.000
Intestinal metaplasia	0.616^**^	−0.061	1.000	0.220^**^	0.132
	0.000	0.439	.	0.005	0.094
Chronic inflammation	0.054	0.453^**^	0.220^**^	1.000	0.498^**^
	0.495	0.000	0.005	.	0.000
Activity	0.151	0.431^**^	0.132	0.498^**^	1.000
	0.055	0.000	0.094	0.000	.

Among the patients aged 75-79 years and ≥80 years, 17 (13.6%) and 22 (28%) very relevant findings (VRFs) at endoscopy were detected, respectively. These VRFs were significantly different between the two age groups (p = 0.0088). Thirty-nine (19.3%) VRFs at endoscopy were cumulatively detected (Table 1). The complication rate was 5%. Hypotension (1%) and hypoxemia (4%) occurred and were treated successfully. Bleeding, perforation, and procedure-related deaths were not seen.

## Discussion

EGD is frequently used in the increasing elderly population. Older patients are more fragile than younger ones because of multiple age-related chronic diseases and the common use of polypharmacy [7,8]. For these reasons, we must be careful doing endoscopic procedures in the elderly. Previously published studies showed that EGD is a safe procedure in elderly patients if good pre-procedural evaluations, such as a cardiorespiratory examination and an airway assessment, are performed [9-11]. Dyspepsia is a common health problem globally and is seen in approximately 20% of the population. Guidelines suggest that patients ≥60 years of age presenting with dyspepsia should be investigated with upper GI endoscopy to exclude GI neoplasia [12,13]. In this study, the most common EGD indication was dyspepsia, followed by GI bleeding, reflux, anemia, and screening/surveillance. This finding is compatible with the existing literature [6,9,14,15]. In the patients’ endoscopic examinations, gastritis was commonly detected and was compatible with chronic active gastritis, which was detected in 42.6% of the patients in pathology reports.

The prevalence of malignancies increased with aging in our results. Among the patients aged 75-79 years and ≥80 years, endoscopic diagnoses of esophageal and gastric malignancies were observed as 6.4% and 18%, respectively. This result is compatible with other studies [2,6,9,16]. Our cumulative diagnostic yield was 19.3% according to VRF, and it increased with age. Our results and literature showed that the yield of EGD is high in elderly patients. We should not give up due to advanced age when making decisions regarding EGD in elderly patients [2,3,6,9,16].

*H. pylori* infection causes active chronic gastritis and plays an important role in the development of atrophic gastritis, as described in Correa’s hypothesis, and gastric atrophy has been recognized as a precancerous condition. Patients with histological intestinal metaplasia and severe atrophy have an increased risk of developing gastric cancer. Significant improvements in gastric atrophy and intestinal metaplasia after the eradication of *H. pylori* may reduce the risk of gastric cancer [17,18]. However, in Grgov et al.’s study, a negative correlation was observed between the frequency of atrophy and *H. pylori*, which is consistent with our results [19]. This conclusion may have been reached because *H. pylori* causes atrophy but does not colonize atrophic areas.

Our complication rate was 5%, and we did not detect major complications. In our hospital, we don’t apply emergency endoscopic intervention and advanced therapeutic procedures. So, this may relate to our low complication rate. Our study and the literature showed that EGD is a safe procedure in elderly patients. We performed an assessment of each patient’s cardiopulmonary status and comorbid conditions and modified the use of sedation or avoided sedation to reduce complications during endoscopic procedures. The diagnostic use of EGD in elderly patients is not associated with high complication rates [3,9,16,20,21].

Our study has certain limitations. It is a retrospective, single-center, and low‐volume study. Our center is a secondary care hospital, and we don’t apply advanced endoscopic interventions. We didn’t know our patients’ medications, previous *H. pylori* eradication histories, or comorbidity statuses. These conditions may affect our results and the complication rate of procedures.

## Conclusions

The application of EGD over the age of 75 is increasing with the expansion in the older population. After pre-procedural evaluation, we must be careful while performing endoscopic procedures in the elderly because of multiple age-related chronic diseases and the common use of polypharmacy. This present study showed that EGD is a safe procedure with a high diagnostic yield in patients aged 75 years and older.
